# Periplocin inhibits the growth of pancreatic cancer by inducing apoptosis via AMPK‐mTOR signaling

**DOI:** 10.1002/cam4.3611

**Published:** 2020-11-24

**Authors:** Gangyin Xie, Linxiao Sun, Yonglin Li, Bicheng Chen, Cheng Wang

**Affiliations:** ^1^ Key Laboratory of Diagnosis and Treatment of Severe Hepato‐Pancreatic Diseases of Zhejiang Province Zhejiang Provincial Top Key Discipline in Surgery The First Affiliated Hospital of Wenzhou Medical University Wenzhou Zhejiang China

**Keywords:** apoptosis, pancreatic cancer, Periplocin, proliferation

## Abstract

**Background:**

Periplocin is a monomeric compound that exhibits anti‐tumor activities. It is extracted from Cortex Periplocae.

**Objective:**

This study aimed at determining the effect of periplocin treatment on the apoptosis and proliferation of human pancreatic cancer cells, and to elucidate on its mechanisms of action.

**Methods:**

PANC1 and cfpac1 cells were treated with periplocin. Cell proliferation was detected by RTCA, Ki67 immunofluorescence, and a clonogenic assay. The transwell assay was used to examine cell migration and invasion functions. The expression of apoptosis‐associated proteins was detected by flow cytometry and western blotting. Total RNA was extracted from the treated and untreated group of PANC1 cells for RNA‐seq detection and analysis. Differentially expressed genes were screened for GO biological process and KEGG pathway analysis. Finally, CFPAC1 cells were subcutaneously inoculated into BALB / c nude mice to assess tumor growth.

**Results:**

Periplocin inhibited the proliferation of PANC1 and CFPAC1 cells and induced their apoptosis by activating the AMPK/mTOR pathway and inhibiting p70 S6K. It also attenuated the cell migration, invasion, and inhibited the growth of cfpac1 xenografts in nude mice.

**Conclusions:**

Periplocin inhibits human pancreatic cancer cell proliferation and induces their apoptosis by activating the AMPK / mTOR pathway.

## Graphical abstract

In this work, we evaluated the effect of Periplocin on pancreatic cancer growth and tried to clarify its molecular mechanism.

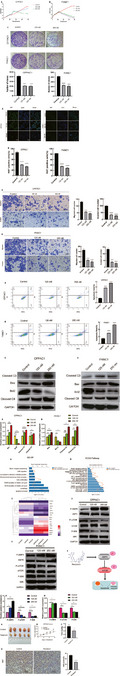

## 1 INTRODUCTION

Cortex periplocae is a traditional herbal medicine with antiradiation, antitumor, anti‐inflammatory, and immune function enhancement effects.^1^ Periplocin is a monomer compound with antitumor activities that is isolated and purified from Cortex periplocae. Periplocin activates the β‐catenin/TCF signaling pathway. In human colon cancer SW480 cells and nude mouse intraperitoneal tumor models, intraperitoneal administration of periplocin (30 mg/kg bw/day) for 12 days inhibited colon cancer growth and induced their apoptosis.^2^ It inhibits lung cancer growth and induces apoptosis by blocking the AKT/ERK signaling pathway.^3^ In normal cells, it exhibits low toxicity. Periplocin inhibits cell viability of human gastric cancer cells through the ERK1 / 2‐EGR1 pathway, thereby inducing cell apoptosis.^4^ In addition, it was shown to inhibit growth while inducing apoptosis by altering the expression of death receptors in gastric cancer SW‐872 cells and initiating DNA double‐strand breaks.^5^ In two mucinous fibroblastic sarcoma (MFS) cell lines (MUG‐Myx2a and MUG‐Myx2b), periplocin mediated apoptosis and cell cycle arrest through the ERK/p38/JNK pathway.^6^ However, the effects of Periplocin in pancreatic cancer have not been established. Therefore, we aimed at determining the effects of periplocin on pancreatic cancer cells as well as its molecular mechanisms of action.

Globally, pancreatic cancer is one of the leading causes of cancer mortalities.^7^ In 2012, approximately 338 000 new pancreatic cancer cases were globally diagnosed. In addition, the mortality rate was almost equal to the incidence rate.^8^ Pancreatic cancer incidences and mortality rates in China are exhibiting an annual upward trajectory.^9^ The prognosis is low, and the 5‐year survival rate is about 5%. Gemcitabine is the primary therapeutic option for pancreatic cancer. It also improves the overall survival rate of very few patients.^10^ Reduced chemotherapeutic efficacies could be attributed to pancreatic cancer drug resistance. It is, therefore, important to develop new medicines for effective targeted therapy.

Many signaling pathways, such as AMPK and mTOR signaling pathways, play an essential role in the progression of pancreatic cancer cells. Studies have shown that AMPK is also important in regulating cell survival and death.^11^ AMPK activation promotes cancer cell death by regulating downstream target proteins.^12^, ^13^, ^14^, ^15^ The mTOR pathway is also involved in various cellular processes, such as protein synthesis,^16^ cell proliferation, and apoptosis.^17^ Many traditional cytotoxic chemicals and natural compounds stimulate AMPK‐dependent death pathways in cancer cells.^18^, ^19^, ^20^ This study aimed at determining the effect of periplocin on pancreatic cancer cell growth and apoptosis. We also explored the molecular mechanisms of its antipancreatic cancer effects.

## 2 MATERIALS AND METHODS

### 2.1 Reagents and antibodies

Periplocin was purchased from Shanghai Yuanye Biotechnology Co. Ltd. DAPI solution was purchased from Solarbio. Cleaved caspase 3, cleaved caspase 8, Bax, Bcl‐2, Ki67, GAPDH antibody, p‐AMPK (Thr172), AMPK, p‐mTOR (Ser2448), mTOR, P‐70S6K (Thr389), and S6K were purchased from Cell Signaling Company. Compound C was purchased from Selleck Chemicals. The BCA protein assay reagent kit and enhanced chemiluminescent (ECL) plus reagent kit were obtained from Pierce (Pierce Biotech).

### 2.2 Cell lines and cell culture

Human PANC1 and CFPAC1 pancreatic cancer cell lines were purchased from the American Type Culture Collection (ATCC). The cell lines were tested for mycoplasma contamination. They were then cultured in a DMEM medium that was supplemented by 10% fetal bovine serum (FBS), streptomycin (100 U/ml), and penicillin (100 U/ml). They were incubated at 37°C in 5% carbon dioxide.

### 2.3 Cytotoxicity assays

The RTCA analysis method was used to determine the viability of pancreatic cancer cells treated with periplocin. The xCELLigence RTCA analyzer has a workstation in the CO_2_ incubator while the control unit has RTCA software (ACEA Biosciences) outside the carbon dioxide incubator. To measure the cytotoxic response of cells in real‐time, they were seeded on embedded 16‐well microplates (E‐plates; Roche Diagnostics). For PANC1 and CFPAC1 cells, the seeding density was 5.0 × 10^3^ cells/well. Impedance was recorded every 15 min. After 20 h of inoculation, 0, 125, and 250 nm periplocin were added to the PANC1 and CFPAC1 cultures, respectively. Between 0 and 72 h at 200 µl is used for all incubations. Cell viability was evaluated by the RTCA‐DP software (Roche Diagnostics GmbH).^18^

### 2.4 Clone formation assay

PANC1 and CFPAC1 cells were inoculated into a six‐well plate at a concentration of 1000 cells/well. They were then treated with periplocin at 0, 125, and 250 nm, respectively, for 24 h. CFPAC1 cells were treated with 250 nm Periplocin and Compound C (5 μm) for 24 h. After 14 days of culture, cell clone formation was checked by staining with crystal violet at room temperature for 15 min.

### 2.5 Immunofluorescence staining analysis

Coverslip cells were washed twice using PBS, fixed with 4% PFA for 15 min at 37°C, and permeabilized with 0.25% Triton X‐100 for 15 min at room temperature. Then, cells were washed twice using PBS and blocked with 3% BSA for 30 min. They were then stained with a primary antibody (ki67) (1: 200) at 4°C overnight. After staining, they were cultured with goat anti‐rabbit secondary antibodies coupled with Alexa 488 (Jackson ImmunoResearch), and incubated at 37°C for 1 h. DAPI nuclear staining was then done for 3 min and the slides observed using a fluorescence microscope (Olympus).

### 2.6 Transwell migration and invasion assay

The cells in a medium containing 1% fetal bovine serum were inoculated onto a matrigel coating (8 μm pore size; HTS ™ FluoroBlok invasion system, BD Biosciences) and an uncoated polycarbonate membrane filter (8 μm pore size; Corning). The lower chamber was filled with a medium containing 20% fetal bovine serum. After 24 and 48 h, the cells that migrated through the membrane and attached to the membrane's bottom were fixed and stained with crystal violet for 15 min. Five random field images (magnification; ×200) were captured from each film. The degree of migration and invasion was expressed as the average number of cells in each microscopic field. All experiments were done in triplicate.

### 2.7 low cytometric analysis of apoptosis

Annexin V‐FITC Apoptosis Detection Kit (BD) was used to evaluate the percentage of apoptotic cells. The PANC1 and CFPAC1 cells were treated with periplocin at 0, 125, and 250 nm for 24 h, respectively. The 5 × 10^5^‐treated cells were centrifuged and resuspended in 1 × cold binding buffer. Five μl Annexin V‐FITC and 5 μl propidium iodide (PI) were added and incubated at room temperature in the dark for 15 min. The Annexin V / PI staining method was performed according to the kit manufacturer's protocol (BD Biosciences). Data collection and analysis were performed on the FC500 MPL system (Beckman Coulter).

### 2.8 RNA‐seq and pathway enrichment analysis

Total RNA was extracted from the 0 and 125 nm Periplocin 24‐h treatment groups of PANC1 cells using TRIzol reagent (Invitrogen). The extracted RNA samples were transported to BGI (Shenzhen, China) for further RNA‐seq detection and analysis using the BGISEQ‐500 sequencer. Pathway analysis of differentially expressed genes (DEG) was performed based on the KEGG database. And Gene Ontology function enrichment. The obtained data was analyzed through the BGI network platform (http://report.bgi.com).

### 2.9 Western blot analysis

The treated cells were obtained and resuspended in cell lysate (Thermo) on ice for at least 30 min. The cell lysate was centrifuged at 12 000 × *g* for 10 min at 4°C. The supernatant was obtained, mixed with 5 × loading buffer, and boiled for 10 min. After boiling, the resulting supernatant containing total protein was quantified using the BCA protein evaluation kit (Beyotime P0012). An 8%, 10%, or 15% polyacrylamide gel (depending on the protein's molecular size to be analyzed) was used to separate a 50 µg total protein sample. The separated sample was transferred to a PVDF membrane (Millipore). The membrane was blocked using 5% skimmed milk and incubated with primary antibodies (1: 1000 in TBST) of cleaved caspase 3, cleaved caspase 8,Bax, Bcl‐2, P‐AMPK, P‐mTOR, p‐p70s6k, and GAPDH. Secondary antibodies coupled with horseradish peroxidase were then introduced into the membrane. The membrane was then incubated. Enhanced chemiluminescence (ECL) reagent (Pierce, Thermo Fisher Scientific) was used to detect protein expression.

### 2.10 Xenograft mouse models

All animal care, use, and experimental protocols were approved by the animal experiment ethics inspection label of the Experimental Animal Center of Wenzhou Medical University. To induce subcutaneous tumor growths, 2 × 10^6^ CFPAC1 cells were subcutaneously injected into the right dorsal midline of BALB / c nude mice (4–6 weeks old). Once the tumor reached ~50 mm^3^ on day 7, nude mice were randomly distributed into four groups (n = 5 nude mice/group). Every 2 days, mice were intraperitoneally injected with PBS (200 ul) and periplocin (15 mg/kg). The length and width of the tumors were measured after every 3 days and tumor volumes calculated using the equation: V = (length × width^2^) /2.

### 2.11 Immunohistochemistry

Immunohistochemistry (IHC) staining was performed to detect ki67 protein expression in tumor xenografts. The formalin‐fixed paraffin‐embedded tissue was cut into 5 µm pieces, dewaxed in xylene, and rehydrated in graded ethanol. Antigenic treatment was performed by placing the slides in a microwave oven for 5 min at high temperature and repeating it two to three times. After treatment with 3% H_2_O_2_ for 10 min, the tissue sections were blocked with 5% normal goat serum. They were first incubated with a primary antibody (ki67) (1: 200) at 4°C overnight, and then, with EnVision Polymer‐HRP secondary antibody at room temperature (Dako, Glostrup, Denmark) for 1 h. After applying the DAB chromogen, tissue sections were stained with hematoxylin, dehydrated, and fixed.

### 2.12 Statistical analysis

Data analysis was performed by the GraphPad Prism 8 software (Graph Pad Software). The cell index (CI) of the real‐time dynamic cytotoxicity assessment (N = 3) was automatically calculated by the RTCA software package 1.2 of the RTCA system. Quantitative data were calculated on the stable line N = 3 ± SD (dashed line) and averaged. The results are expressed as mean ±SD. The Student's t‐test and one‐way analysis of variance were used to determine statistical significance. *p* ≤ 0.05 was set as the threshold for statistical significance.

## 3 RESULTS

### 3.1 Periplocin inhibited growth in PDAC cell lines

**FIGURE 1:**
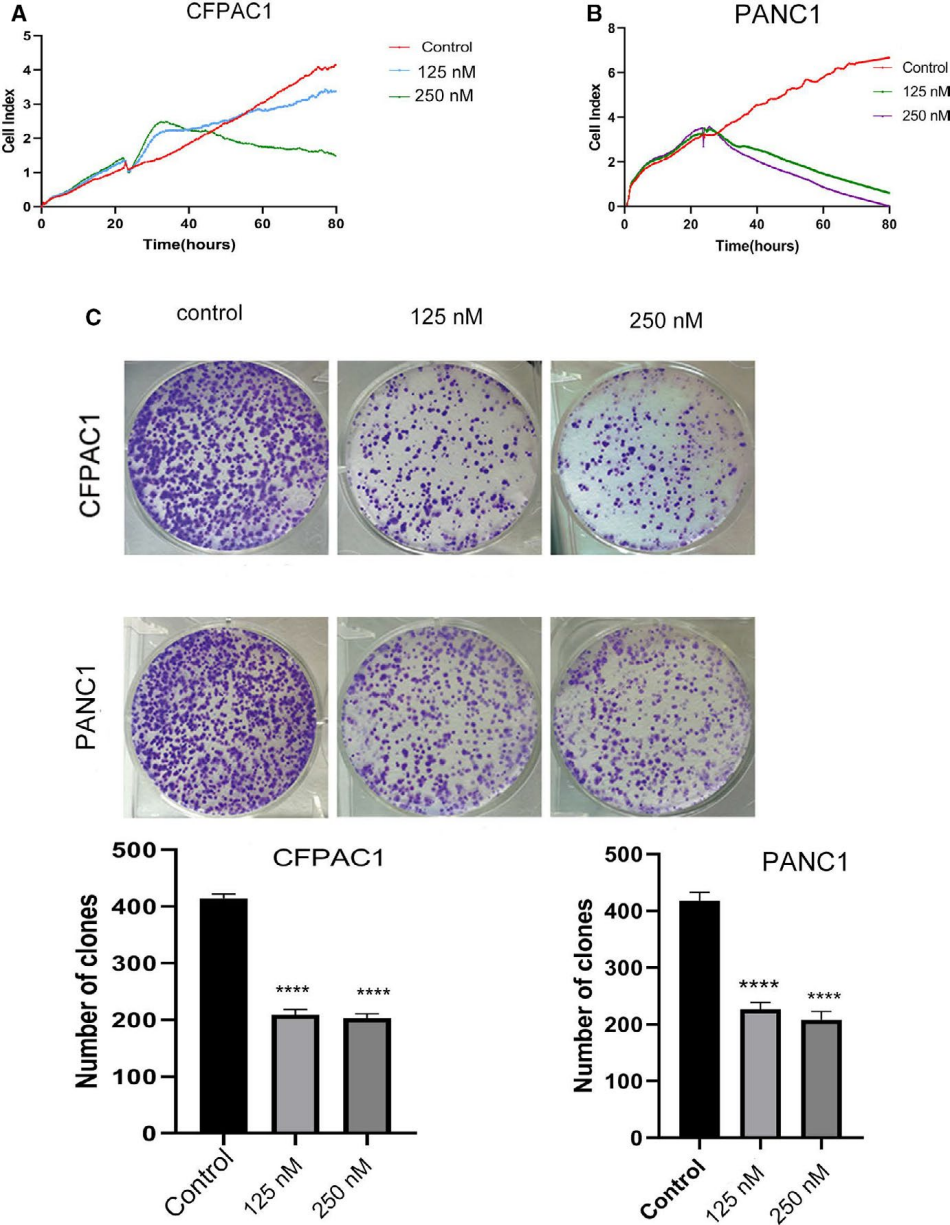
Proliferation inhibition by Periplocin in PDAC cells. A and B, CFPAC1 and PANC1 cells were treated with 0,125, and 250 nm periplocin for 0–72 h, respectively. Cell viability was determined using an RTCA assay. C, CFPAC1 and PANC1 cells were seeded in six‐well plates and treated with 0, 125, and 250 nm periplocin, respectively. Colony formation was assessed by crystal violet staining after 14 days of culture. The results are the mean ±SD of independent experiments performed in triplicate. *p* ≤ 0.05 was considered to be statistically significant, * *p* < 0.05, ** *p* < 0.01, *** *p* < 0.001, **** *p* < 0.0001 versus the control

After treatment with periplocin, the cell survival index decreased in a concentration‐dependent manner (Figure 1A,B). In addition, periplocin‐treated PANC1 and CFPAC1 cells exhibited fewer colonies than control cells (Figure 1C).

**FIGURE 2:**
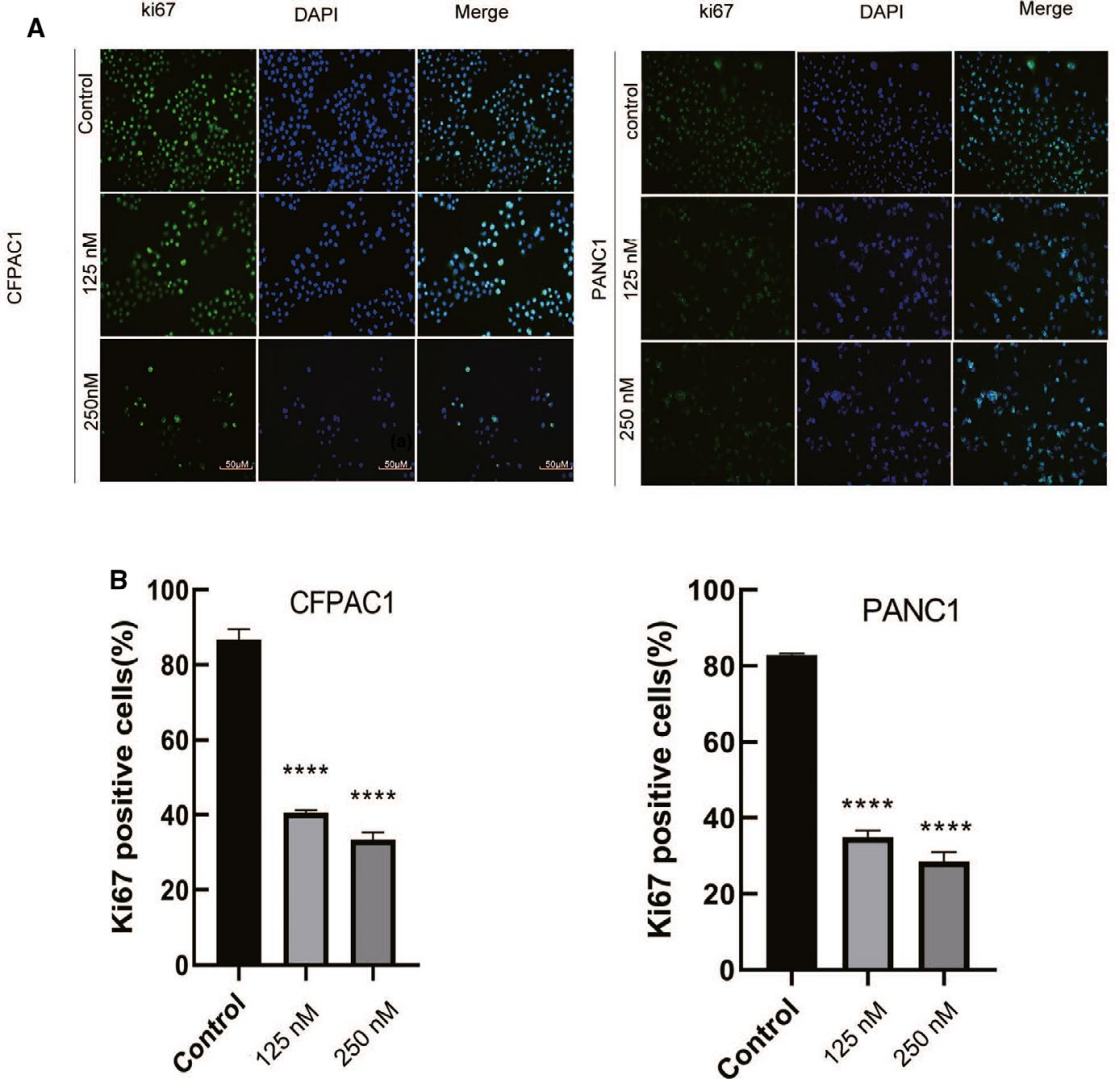
Periplocin reduces the expression of Ki67 Immunofluorescence. A, After respectively treating CFPAC1 and PANC1 cells with 0,125, and 250 nm periplocin for 24 h, Ki67 Immunofluorescence showed cell proliferation at different periplocin concentrations. B, Ki67 Immunofluorescence and quantitative analysis. Scale bar, 50 μm. The results are expressed as the mean ±SD of independent experiments performed in triplicate. *p* ≤ 0.05 was considered to be statistically significant, * *p* < 0.05, ** *p* < 0.01, *** *p* < 0.001, **** *p* < 0.0001 versus the control

We also determined whether periplocin decreased the proliferation markers of human pancreatic cancer cell (ki67). Correspondingly, Ki67 Immunofluorescence and analysis showed that the ki67 green fluorescence in the tumor cell‐treated group was significantly reduced compared to the untreated group (Figure 2A,B ).

### 3.2 Periplocin inhibits pancreatic cancer cell migration and invasion

**FIGURE 3:**
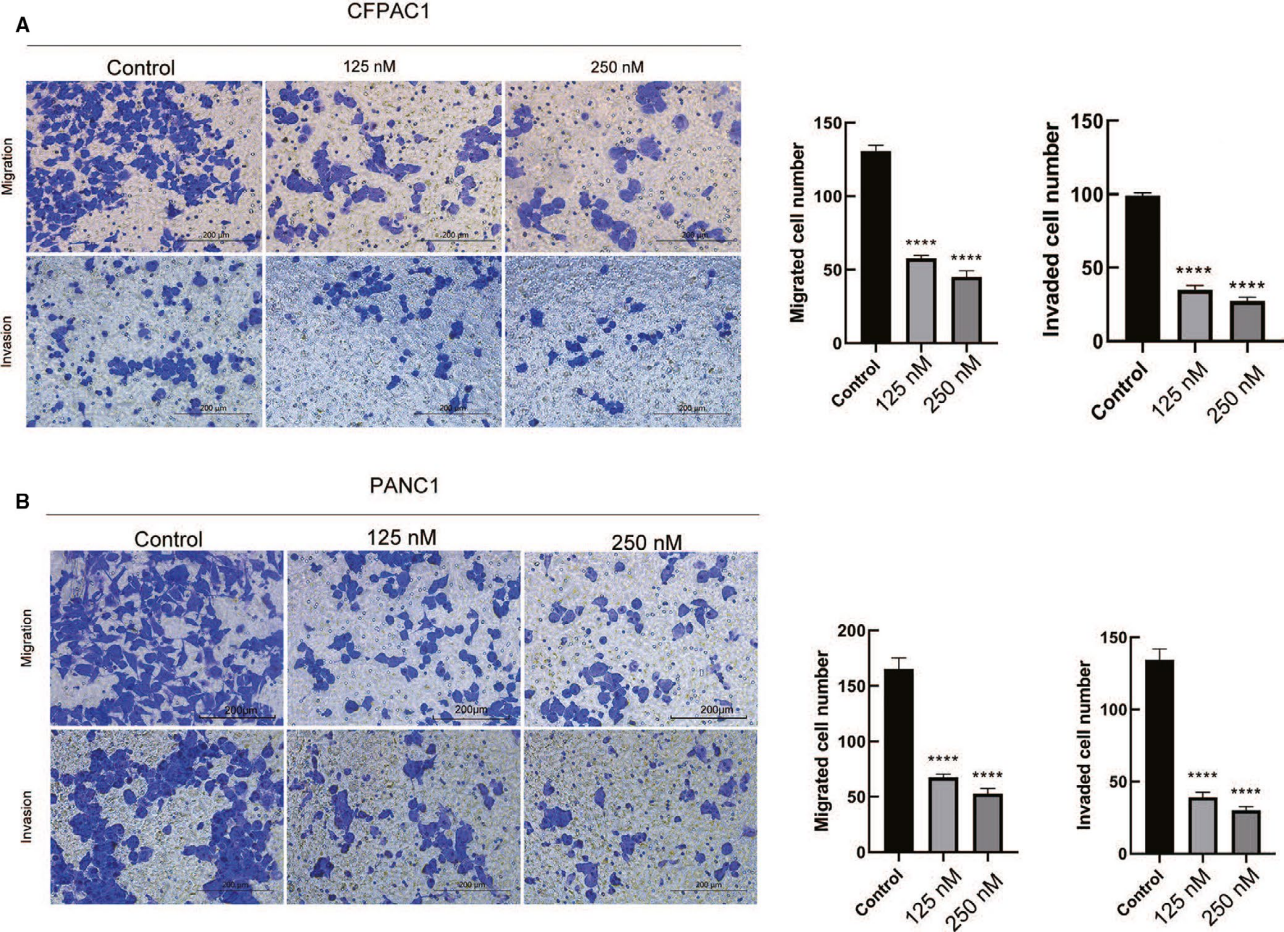
Periplocin attenuated the migration and invasive ability of human pancreatic cancer cells. A, CFPAC1 cells were treated with 0,125, and 250 nm periplocin for 24 h for transwell migration experiments and for 48 h for transwell invasion experiments. In the transwell invasion experiment, the cells in the medium containing 5% fetal bovine serum were seeded into the upper cavity of a Matrigel‐coated polycarbonate membrane filter (8 μm pore size). B, PANC1 cells were treated with 0,125, and 250 nm periplocin for 24 h for transwell migration experiments, and 48 h for transwell invasion experiments. Scale bar, 200 μm. The results are the mean ±SD of independent experiments performed in triplicate. *p* ≤ 0.05 was considered to be statistically significant, * *p* < 0.05, ** *p* < 0.01, *** *p* < 0.001, **** *p* < 0.0001 versus the control

There was a significant reduction in cell migration and invasion in periplocin‐treated CFPAC1 and PANC1 cells, respectively (Figure 3A,B).

### 3.3 Periplocin induces apoptosis in pancreatic cancer cells

**FIGURE 4:**
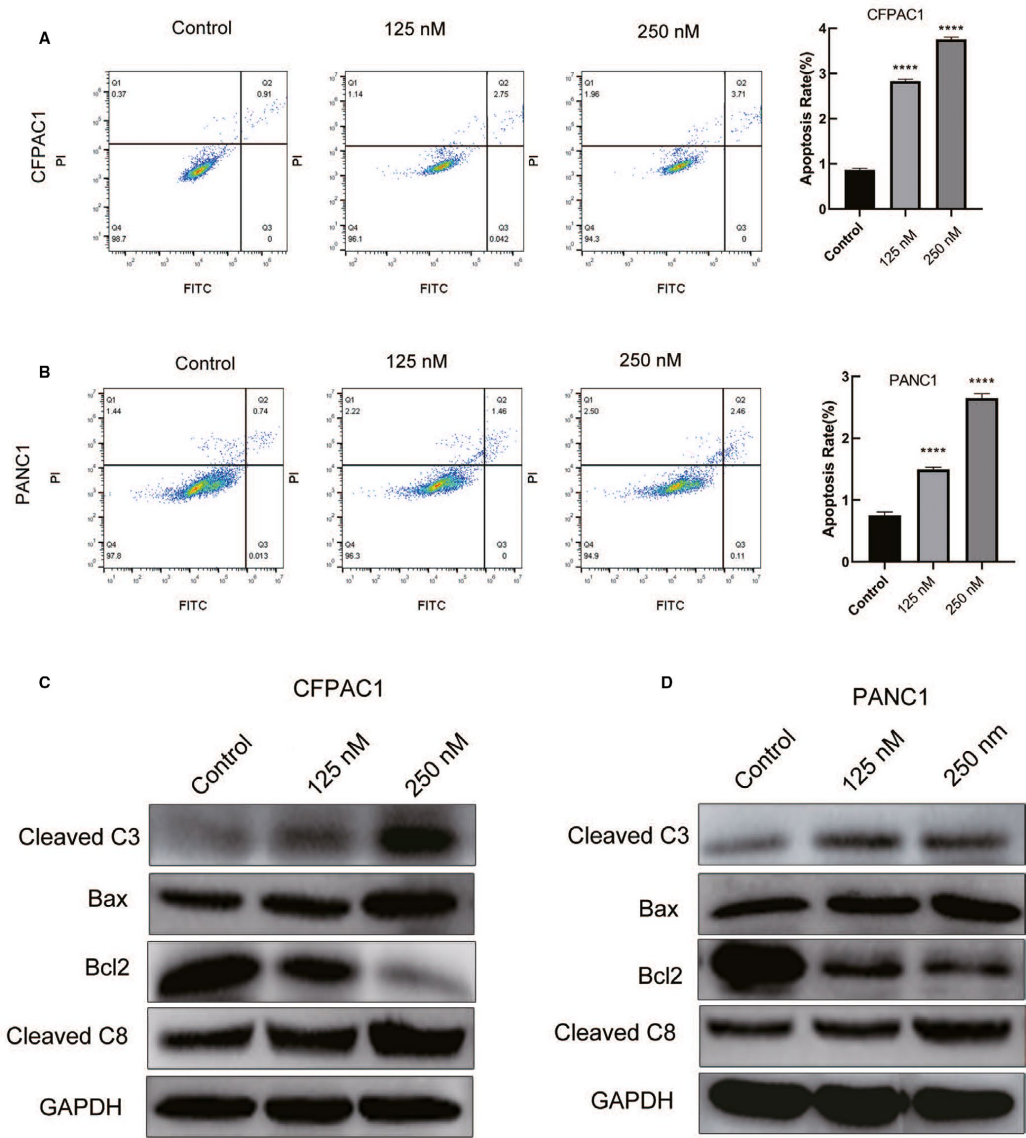
Periplocin induces apoptosis of human pancreatic cancer cells. A and B, CFPAC1 and PANC1 cells were treated with periplocin at 0, 125 nm, and 250 nm for 24 h. The annexin V‐FITC assay was performed to determine the apoptotic rate of cells treated with periplocin at different concentrations. The results were analyzed by flow cytometry. C and D, The proteins expressed by the treated human pancreatic cancer cells were examined by Western blotting. Apoptosis‐associated proteins (cleaved caspase 8, cleaved caspase 3, Bax, and Bcl2) were detected using the corresponding antibodies with GAPDH as the control. The results are presented as the mean ±SD of independent experiments performed in triplicates. *p* ≤ 0.05 was considered to be statistically significant, * *p* < 0.05, ** *p* < 0.01, *** *p* < 0.001, **** *p* < 0.0001 versus the control

**FIGURE 5:**
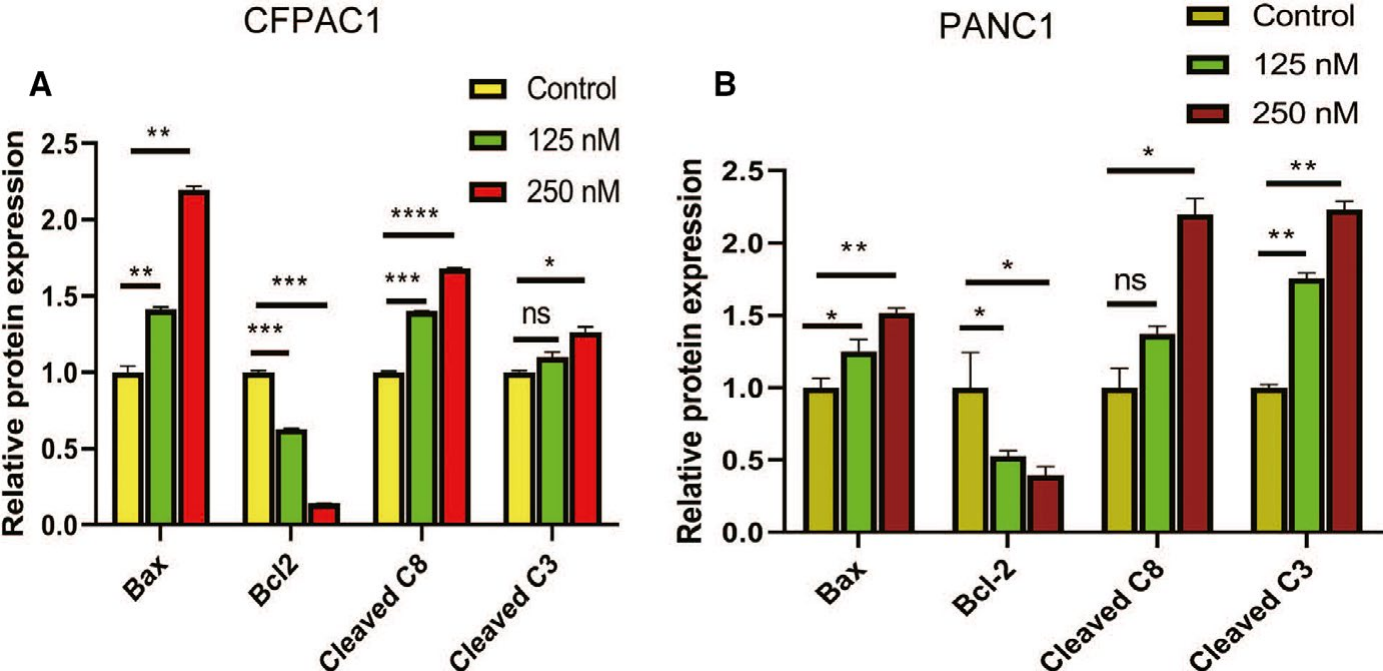
Quantitative analysis of Western blot apoptotic proteins. Data analysis of Figure 4C‐D. Data are presented as the mean ±standard deviation. *p < 0.05, **p < 0.01, n ≥ 3.

As shown in Figure 4A,B, the apoptotic rate was significantly high in human pancreatic cancer cells treated with periplocin. In addition, periplocin treatment elevated the expression of apoptosis‐associated proteins (Bax,cleaved Caspase‐8 and cleaved Caspase‐3) and decreased the expression of Bcl‐2 (Figure 4C,D). The data analysis of Figure 4C,D is shown in Figure 5A,B. In summary, periplocin induced apoptosis in PANC1 and CFPAC1 cells.

### 3.4 Periplocin regulates the AMPK/mTOR signaling pathways in human pancreatic cancer

**FIGURE 6:**
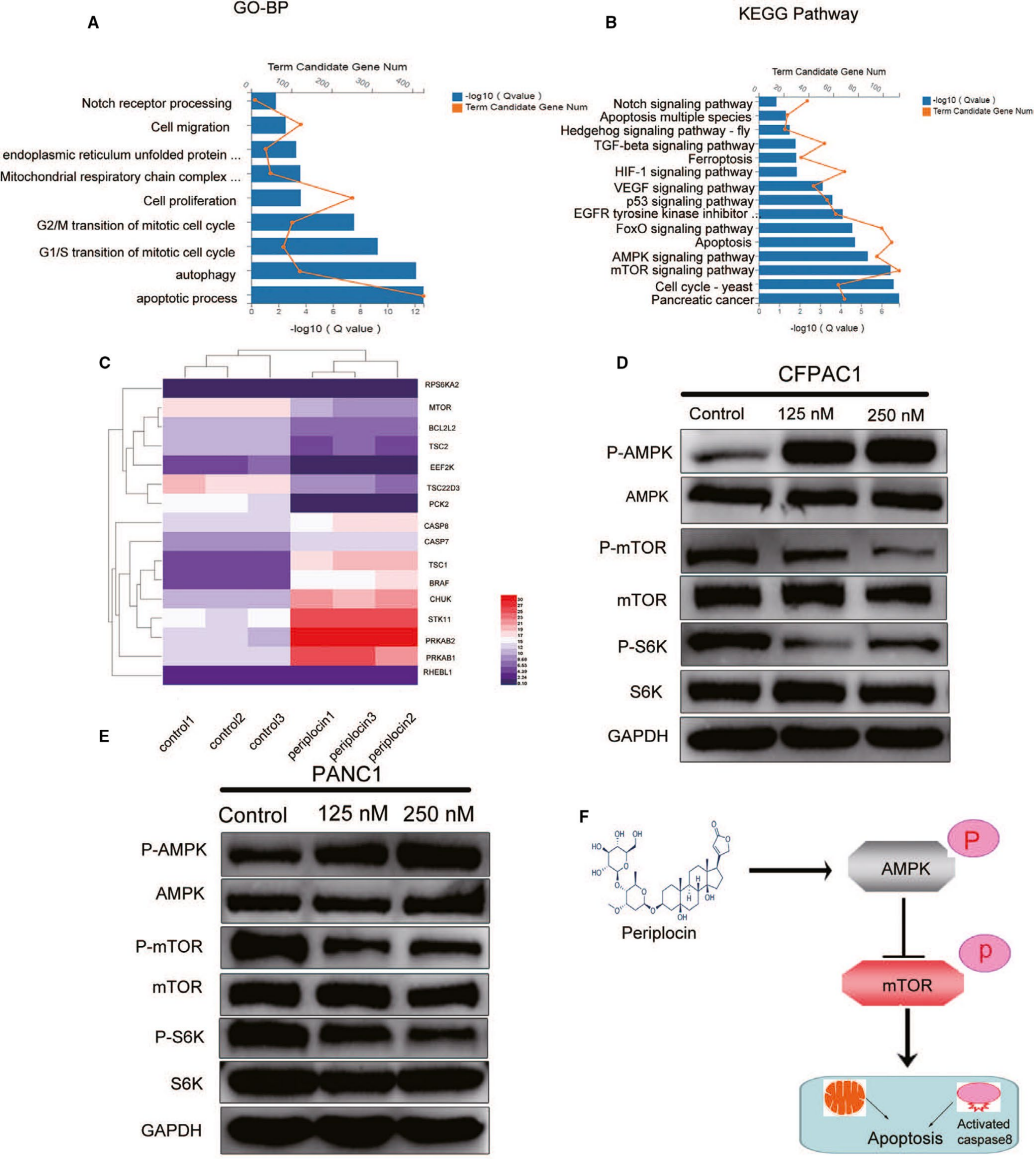
Periplocin regulated the AMPK/mTOR signaling pathways in pancreatic cancer. A and B, Gene ontology (GO) biological process and KEGG pathways analysis based on the RNA‐seq results for control versus Periplocin in PANC1 cells. *p*‐value was corrected by FDR, and the FDR <0.01 was considered significantly enriched. C, The hierarchical clustering of targets in the AMPK/mTOR signaling pathway depicted as a heat map. Downregulated genes are shown in blue and upregulated genes in red. D and E, Western blot analysis showed the expression changes of critical molecules in the AMPK/mTOR signaling pathway in PANC1 and CFPAC1 cells. F, Proposed mechanism of periplocin‐induced apoptosis in pancreatic cancer cells. Periplocin inhibits mTOR signaling by activating the AMPK signaling pathway, thus, inhibiting s6 k and exerting anti‐tumor effects

**FIGURE 7:**
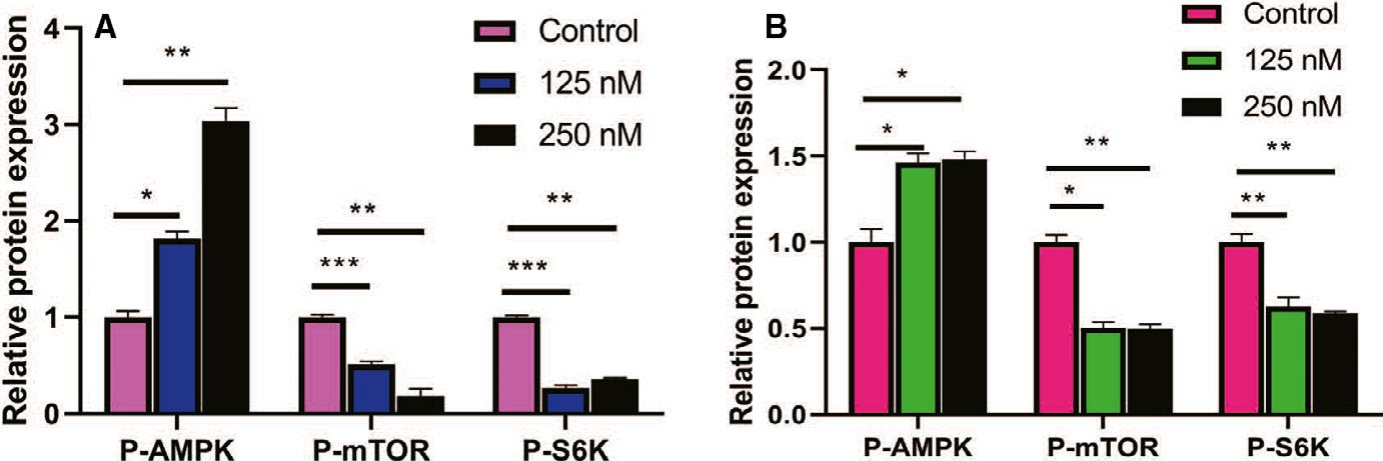
Quantitative analysis of Western blot pathway proteins. Data analysis of Figure 6D–E. Data are presented as the mean ±standard deviation. *p < 0.05, **p < 0.01, n ≥ 3

By performing a t‐test with a critical (q) value of <0.05 and FC>0.5, DEG was selected. Functional enrichment analysis were then performed on the co‐expressed DEG. In the GO biological process, representative GO terms included apoptosis process, autophagy, cell proliferation, Notch receptor processing, among others (Figure 6A). The KEGG pathway analysis clustered these genes into several pathways (Figure 6B). The AMPK/mTOR signaling pathway exhibited a strong correlation with tumor growth and proliferation. The heat map (Figure 6C) highlights 16 representative genes differentially expressed in the AMPK/mTOR signaling pathway and apoptosis. As shown in Figure 6D,E Western blot analysis confirmed the heat map results in the AMPK/mTOR signaling pathway, which upregulated P‐AMPK and downregulated the expression of P‐mTOR and P‐S6K proteins. Periplocin was found to exert its antitumor effects by inhibiting mTOR signaling through the activation of the AMPK signaling pathway. These effects inhibit S6K and, therefore, exerting antitumor effects (Figures 6F and 7A,B). The data analysis of Figure 6D,E are presented in Figure 7A,B.

### 3.5 Periplocin inhibits the growth of CFPAC1 xenograft mouse models

**FIGURE 8:**
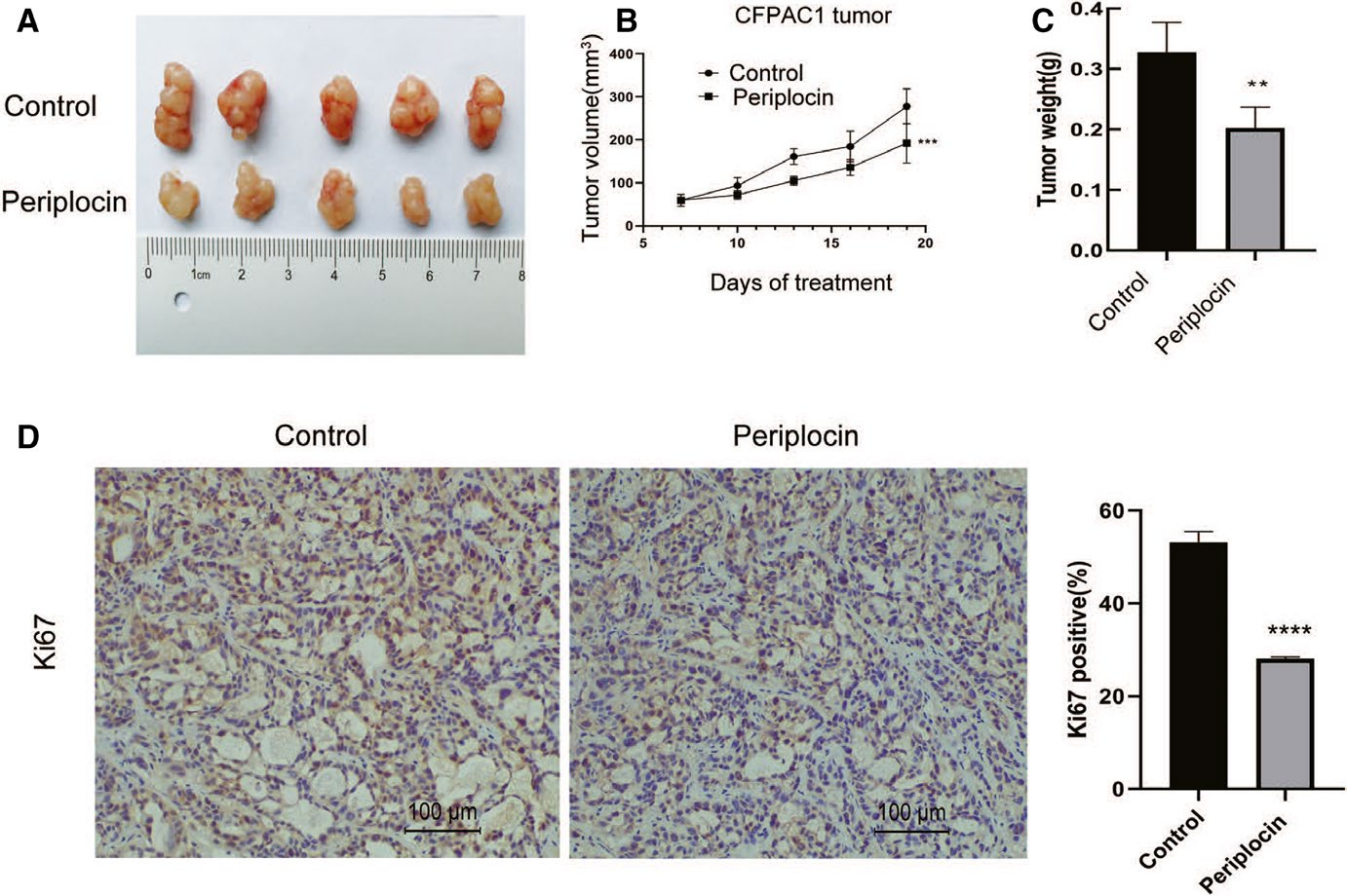
Periplocin inhibits the growth of CFPAC1 xenografts in nude mice. CFPAC1 cells were inoculated into nude mice to establish subcutaneous xenograft tumors. The dissected tumors were photographed. A, Representative macroscopic views showed tumors in indicated groups. B, Tumor volumes were monitored every 3 days after indicated treatments. C, Dissected tumor weights were measured. D, Representative images of IHC staining. Scale bar, 100 μm. Data are presented as means ±SD (* *p* < 0.05,** *p* < 0.01,*** *p* < 0.001), n ≥ 3

Figure 8A–C shows the tumor volumes as measured after every three days, and the tumor weights as measured at the end of the study. It is shown that periplocin inhibited the growth of CFPAC1 xenograft tumors in nude mice. In addition, periplocin was associated with reduced expression of Ki67 (Figure 8D). These findings were consistent with the in vitro results.

## 4 DISCUSSION

In this study, RNA‐seq differential gene Kegg pathway analysis and Immunoblotting of PANC1 cells treated with periplocin showed that the AMPK/mTOR pathway was activated while p70 S6 K was inhibited. The growth of pancreatic cancer cells was also inhibited. Furthermore, periplocin activated AMPK/mTOR to attenuate the proliferation of human pancreatic cancer cells. MTOR and AMPK were used as low energy and high energy sensors, respectively. They can be activated by glucose starvation and sufficient nutrients to promote cell growth and inhibition.^21^, ^22^ Forced AMPK activation inhibits the mammalian target of rapamycin (mTOR) complex 1 (mTORC1)^23^ and the downstream S6K protein synthesis. These effects suppress tumor growth. This finding is consistent with those reported by previous studies. For example, berberine has been shown to inhibit proliferation, migration, and invasion by targeting the AMPK/HNF4α/WNT5A pathway in gastric cancer cells.^24^ It has also been established that AMPK and death receptor five are involved in the apoptosis of hepatocellular carcinoma cells (HCC) and are induced by TRAIL and Berberine combined therapy.^25^ Yuanhuacine (YC) was found to significantly enhance the AMP‐activated protein kinase (AMPK) signaling pathway in H1993 human NSCLC cells while inhibiting the downstream signaling pathway mediated by mTORC2, thereby suppressing cell proliferation.^26^ The natural compound (Tanshinone IIA) also induces autophagic cell death by activating AMPK and ERK in leukemia KBM‐5 cells and inhibiting mTOR and p70 S6K.^27^ Contrastingly, Panduratin A was shown to induce protective autophagy in melanoma A375 cells by activating AMPK and inhibiting mTOR signal transduction, thereby, conferring an antiapoptotic effect.^28^ It has also been found that RA‐XII inhibits AMPK phosphorylation in HepG2 cells and activates the mTOR/P70S6K pathway. These effects induce HepG2 cell apoptosis and inhibits protective autophagy.^29^ The possible explanation is that the AMPK‐mTOR signaling pathway is involved in the regulation of autophagy. Inhibition or activation of mTOR, therefore, inevitably causes the activation or inhibition of autophagy. Autophagy in cancer cells is conducive for cancer cell proliferation as it exerts antiapoptotic effects. However, excessive autophagy also lead to autophagy‐dependent cell death. The finding that excessive autophagy leads to cell growth inhibition should be further verified. In summary, periplocin inhibits mTOR through AMPK activation, thereby, suppressing the proliferation of pancreatic cancer cells, their migration, and invasion.

We also revealed that periplocin induces pancreatic cancer cell apoptosis through the AMPK/mTOR/S6K pathway. In addition, proapoptotic molecules (Bax and caspase3/8) were activated while the antiapoptotic Bcl‐2 was inhibited. This implies that the apoptosis induced by periplocin is a combination of caspase‐dependent internal and external apoptotic pathways. Programed cell death (PCD) is an orderly process. Apoptosis is a form of programed cell death. Three pathways mediate apoptosis: the mitochondrial pathway, the endoplasmic reticulum pathway, and the death receptor pathway. It has been established that quercetin inhibits S6K by activating the AMPK signaling pathway, thereby inducing bladder cancer cell apoptosis.^30^ Periplocin upregulates death receptors DR4 and DR5 by activating the ERK1 / 2‐EGR1 pathway that induces the apoptosis of gastric cancer cells.^18^ In TRAIL‐resistant human hepatocellular carcinoma cells, periplocin downregulates IAP to promote apoptosis.^31^ It has been shown that S6K is involved in cell growth, cell cycle‐related protein regulation, and translation.^32^, ^33^, ^34^ In addition, S6k is involved in cell apoptosis and is closely related to AKT.^35^ It has not been established whether periplocin induces cell cycle arrest, leads to cell apoptosis, or inhibits phosphorylation targets indirectly by regulating S6K and downstream kinases. Proteomics analysis revealed different interactions between p70‐S6K1 and p54‐S6K2 and found evidence of new targets or regulators of the S6K protein family, such as NCL, NPM1, eIF2α, XRCC6, PARP1, and ILF2/ILF3.^36^ This study elucidates on the S6K interaction network, and may help in understanding the mTOR/S6K pathway. In conclusion, periplocin induces pancreatic cancer cell apoptosis through the S6k pathway.

The rising pancreatic cancer incidences and mortality have necessitated the development of new treatment strategies. Analogs of natural compounds can be used to treat pancreatic cancer.^37^ Periplocin has been shown to inhibit cell proliferation while inducing apoptosis. In addition, it inhibited the growth of the xenograft mouse model of CFPAC1 cells. Therefore, periplocin is a potential alternative for the treatment of human pancreatic cancer.

### 4.1 Conclusion

This study confirmed the inhibitory effects of periplocin on pancreatic cancer cells. By detecting the protein expression levels of important signaling pathways, periplocin was shown to induce caspase dependent apoptosis through the AMPK / mTOR apoptosis signal.

## CONFLICTS OF INTEREST

There are no conflicts of interest to disclose.

## AUTHOR CONTRIBUTIONS

All authors read and approved the manuscript. B.C. and C.W. designed the experiment and analyzed the results. G.X. and L.S. performed the experiment and were responsible for writing the manuscript. Y.L. participated in the experimental procedures and analyzed the data.

## Data Availability

Not applicable.
